# PD-1-CD28 fusion protein strengthens mesothelin-specific TRuC T cells in preclinical solid tumor models

**DOI:** 10.1007/s13402-022-00747-9

**Published:** 2022-11-21

**Authors:** Stefanie Lesch, Alessia Nottebrock, Felicitas Rataj, Constanze Heise, Stefan Endres, Sebastian Kobold

**Affiliations:** 1 Center of Integrated Protein Science Munich (CIPS-M) and Division of Clinical Pharmacology, Department of Medicine IV, Member of the German Center for Lung Research (DZL), University Hospital, Ludwig-Maximilians-Universität München, Munich, Germany; 2 German Center for Translational Cancer Research (DKTK), partner site Munich, Munich, Germany; 3 Einheit Für Klinische Pharmakologie (EKLiP), Helmholtz Zentrum München, Germany, Research Center for Environmental Health (HMGU), Neuherberg, Germany; 4 Division of Clinical Pharmacology, Klinikum der Universität München, Lindwurmstraße 2a, 80337 Munich, Germany

**Keywords:** Adoptive cell transfer, TRuC T cells, PD-1-CD28 fusion protein, Immunosuppression, T cell hypofunction

## Abstract

**Background:**

T cell receptor fusion constructs (TRuC) consist of an antibody-based single chain variable fragment (scFv) fused to a T cell receptor chain (TCR) and allow recognition of cancer cells in an HLA-independent manner. Unlike chimeric antigen receptors (CAR), TRuC are integrated into the TCR complex resulting in a functional chimera with novel specificity, whilst retaining TCR signaling. To further enhance anti-tumor function, we expressed a PD-1-CD28 fusion receptor in TRuC T cells aiming to prevent tumor-induced immune suppression and T cell anergy.

**Methods:**

The activation level of engineered T cells was investigated in co-culture experiments with tumor cells followed by quantification of released cytokines using ELISA. To study T cell-mediated tumor cell lysis in vitro, impedance-based real-time tumor cell killing and LDH release was measured. Finally, two xenograft mouse cancer models were employed to explore the therapeutic potential of engineered T cells.

**Results:**

In co-culture assays, co-expression of PD-1-CD28 enhanced cytokine production of TRuC T cells. This effect was dependent on PD-L1 to PD-1-CD28 interactions, as blockade of PD-L1 amplified IFN-γ production in unmodified TRuC T cells to a greater level compared to TRuC-PD-1-CD28 T cells. In vivo, PD-1-CD28 co-expression supported the anti-tumor efficacy of TRuC T cells in two xenograft mouse cancer models.

**Conclusion:**

Together, these results demonstrate the therapeutic potential of PD-1-CD28 co-expression in TRuC T cells to prevent PD-L1-induced T cell hypofunction.

**Supplementary Information:**

The online version contains supplementary material available at 10.1007/s13402-022-00747-9.

## Introduction

Genetic engineering of T cells has become a major area in the field of cancer immunotherapy [1]. T cells equipped with a CD19-specific chimeric antigen receptor (CAR) have demonstrated impressive therapeutic and curative potential in several B cell malignancies [2]. Therefore, anti-CD19-CAR T cells are part of the standard of care, amongst others, in refractory or relapsed diffuse large B cell lymphoma or acute lymphoblastic leukemia [1, 3]. As more indications for CAR T cells are emerging in hematology, the therapeutic use of T cells, also referred to as adoptive cell therapy (ACT), could not yet deliver convincing results in patients suffering from solid tumors. To extend the successful application of cell therapies beyond hematological malignancies, several novel approaches are studied but still must prove therapeutic benefits in clinical settings. In essence, access to the tumor site, tumor (antigen) heterogeneity and immune suppression are deemed critical hurdles for activity of therapeutic T cells in solid tumors [4]. The currently most promising strategies addressing some of these aspects are: intratumoral delivery of CAR T cells [5], optimized tumor targeting by bispecific CARs [6, 7], combination with immune checkpoint blockade [8], cytokine-secreting CAR T cells [9, 10] and co-expression of chemokine receptors [11–13]. The outcomes of ongoing clinical trials are urgently awaited and will provide important information for the further development of next-generation cell-based cancer therapies.

Although the success of CAR T cells is unchallenged, the synthetic nature of the CAR structure and signaling come with certain limitations of their own [14]. It has been, for example, hypothesized that CAR have disadvantages in signal transduction resulting in low performance when directly compared to TCR in certain situations [15]. Along these lines, using TCR signaling without the shortcomings of HLA restrictions might boost anti-tumor activity by improving downstream signaling and thus T cell function. Synthetic T cell receptor fusion construct (TRuC) represent a novel receptor class which consist of a tumor antigen-specific single chain variable fragment (scFv) fused to the CD3ε subunit [16]. Upon introduction into T cells, the synthetic fusion protein is integrated into the TCR complex to mediate a novel and HLA-independent target specificity [16]. In preclinical models, TRuC T cell were shown to be equivalent or superior to CAR T cells regarding tumor cell lysis and tumor control while producing less cytokines, which could translate in a better safety profile of TRuC T cells in clinical settings, where cytokine release syndrome (CRS) is a severe and frequently observed side effect of ACT [17]. Based on these promising findings, a phase I clinical trial was recently launched with TRuC T cells targeting mesothelin in patients with mesothelin-positive cancers. Preliminary results of the study indicate a manageable safety profile and early signs of activity [18]. While encouraging, clinical responses were limited calling for strategies to enhance TRuC T cell activity.

Like endogenous tumor-infiltrating T cells (TILs) or CAR T cells, TRuC T cells can be negatively impacted by the immune-suppressive tumor microenvironment hampering their anti-tumor efficiency [8]. One of the major inhibitory mechanisms utilized by tumors is the expression of programmed death ligand 1 (PD-L1), which is an indicator of poor prognosis in several solid and hematological malignancies. The interaction of PD-L1 positive tumor cells with programmed death 1 (PD-1) on activated T cells, including CAR or TRuC T cells, induces T cell anergy and exhaustion, blunting therapeutic efficacy [19, 20]. Several strategies have been developed to counteract the PD-L1—PD-1-mediated suppression of T cell function, including blocking monoclonal antibodies [21], PD-1 knockout by CRISPR-Cas9 [22], dominant-negative receptors [23], PD-1-CD28 fusion proteins [24, 25] or anti-PD-1 scFv secreting T cells [26]. We previously pioneered the use of PD1-CD28 fusion proteins [24, 27, 28], and hypothesized that prevention of PD-1-induced suppression might be most effective and safest if applied directly in the T cell product rather than systemically or locally. We thus chose to co-express a PD-1-CD28 fusion protein in TRuC T cells and characterized the additive effect of both receptors in primary human T cells. To mimic the clinical situation of the investigational TRuC and to generate results with direct clinical impact, we designed an anti-mesothelin TRuC, which expressed in T cells mediated efficient T cell activation and tumor cell lysis. Additional expression of PD-1-CD28 shielded TRuC T cells from PD-L1-induced immunosuppression and increased IFN-γ and IL-2 release in the presence of PD-L1 + tumor cells. In mouse xenograft models, PD-1-CD28 co-expression in TRuC T cells enhanced tumor control and survival. Our study, therefore, suggests the therapeutic potential of PD-1-CD28 in preventing hypofunction of TRuC T cells in PD-L1 + solid tumors.

## Material and methods

### Cell lines

The human tumor cell lines SUIT-2, MIA PaCa-2 and MSTO-211H were purchased from ATCC. Cell lines have been modified with retroviruses to express the full-length human mesothelin protein (UniProt entry Q13421) and the full-length human PD-L1 protein (UniProt entry Q9NZQ7). SUIT-2 and MIA PaCa-2 cell lines were grown in DMEM medium with 10% fetal bovine serum (FBS, Life Technologies, USA), 1% penicillin and streptomycin (PS) and 1% L-glutamine (all from PAA, Germany). MSTO-221H cells were grown in RPMI-1640 medium with 10% fetal bovine serum (FBS, Life Technologies, USA), 1% penicillin and streptomycin (PS) and 1% L-glutamine (all from PAA, Germany). All tumor cells were used for experiments in exponential growth phase. 293Vec-Galv and 293Vec-RD114 were a kind gift of Manuel Caruso, Québec, Canada and have previously been previously described [29].

To produce retroviruses, pMP71 vectors (kindly provided by C. Baum, Hannover) carrying the sequence of the relevant receptor were stably introduced in packaging cell lines [11]. Single cell clones were generated and indirectly screened for highest level of virus production by determining transduction efficiency of primary T cells. This method was used to generate the producer cell lines 293Vec-RD114-TRuC and -PD-1-CD28. 293Vec-Galv and 293Vec-RD114 were grown in DMEM with 10% fetal bovine serum (FBS, Life Technologies, USA), 1% penicillin and streptomycin (PS) and 2% L-glutamine (all from PAA, Germany). Primary human T cells were cultured in VLE-RPMI 1640 (Biochrom, Germany) containing 2.5% human serum, 1% PS, 1% L-glutamine, 1% NEAA, 1% sodium pyruvate (TCM). 50 µM β-mercaptoethanol, 10 IU/ml IL-2 and 100 µg/ml IL-15 were added to TCM when culturing the T cells. All cell lines used in experiments were regularly checked for mycoplasma species with the commercial testing kit MycoAlert (Lonza). Authentication of human cell lines by STR DNA profiling analysis was conducted in house.

### Animal experimentation

6- to 10-week-old female NSG mice (NOD.Cg-Prkdcscid Il2rgtm1WjI/SzJ) were purchased from Charles River (Sulzfeld, Germany). The SUIT-2 xenograft model was established by subcutaneous injection of 2 × 10^6^ cells in PBS. For the MSTO-221H tumor model, 10^6^ cells in Matrigel (BD Sciences, Heidelberg, Germany) were implanted through subcutaneous injection. 10^7^ T cells were given intravenously as indicated. Animals were housed in specific pathogen-free facilities at the Klinikum der Universität München. All animal experiments were approved by the local regulatory agency (Regierung von Oberbayern). Tumor measurements and endpoints were registered by an observer blinded to the treatment groups as previously defined[15]. For ethical reasons, endpoints of survival studies were defined as tumor ulceration, tumor sizes exciding 15 mm in any dimension, weight loss above 15% or clinical signs of distress. Manifestation of graft-versus-host disease (GvHD) judged by decreased mobility, general weakness, hunched posture or ungroomed hair defined an additional endpoint regardless of tumor burden.

### Retroviral transduction and T cell expansion

The TRuC construct was generated by tethering the scFv of the mesothelin-targeting monoclonal antibody SS1 to the TCR ε-subunit via the flexible liner (GGGGS) × 3 as described previously [5]. As a control, a second-generation CAR comprising the SS1-derived scFv coupled to CD28 and CD3ζ has been used. The PD-1-CD28 fusion protein consists of the human PD-1 extracellular and transmembrane domain fused to the intracellular domain of human CD28. Expression of the transgenes was verified by flow cytometry using PE-labelled recombinant human mesothelin (MSN-H526x, Acro Biosystems, USA) and anti-human PD-1 (clone EH12.2H7, Biolegend, USA). Transduction and expansion of primary human T cells was carried out following a previously described protocol [15].

### Co-culture experiments

T cells were incubated with tumor cell lines at indicated effector-to-target ratios. Following a 24-h co-culture, supernatants were used to quantify tumor cell lysis using the CytoTox 96® Non-Radioactive Cytotoxicity Assay (Promega, USA) according to the manufacturer’s protocol. IFN-γ and IL-2 was quantified by enzyme-linked immunosorbent assay (ELISA, BD bioscience). For blocking assays, 10 µg/ml anti-human PD-L1 (clone 29E.2A3, Biolegend, USA) and appropriate isotype control were added to the co-culture.

### xCELLigence assays

Real-time tumor cell killing was studied by using the impedance-based xCELLigence system (ACEA Bioscience, USA). 10^4^ tumor cells were seeded per well in a 96-well plate. When the tumor cells reached an index of 0.5–1.0, 10^5^ T cells transduced with the indicated receptor were added. Impedance values were quantified for up to 110 h every 20 min.

### Statistical analysis

Statistical evaluation was performed by using GraphPad Prism software V9 (San Diego, CA, USA). Differences between experimental conditions were analyzed as described in figure *P* values < 0.05 were considered to be significant. Data are shown as mean values SEM of a minimum of three biological replicates or independent experiments, as indicated.

## Results

### Design and expression of MSLN-TRuC and MSLN-TRuC + PD-1-CD28 in primary human T cells

**Fig. 1:**
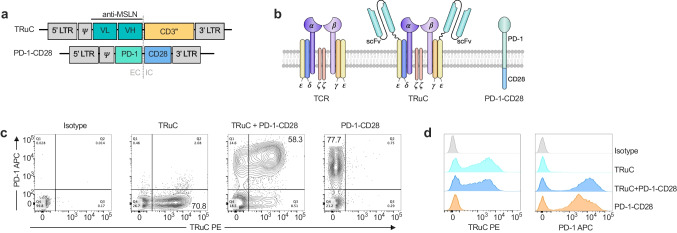
Design and expression of TRuC and PD-1-CD28. **a, b**. The anti-mesothelin scFv was fused to CD3ε assembling a mesothelin-specific TRuC when expressed in human primary T cells. The PD-1-CD28 construct consists of an extracellular PD-1 domain and an intracellular CD28 domain. **c, d**. Following retroviral transduction, all constructs were stably expressed in human T cells as confirmed by flow cytometry. TRuC + PD-1-CD28 T cells were generated by transducing cells with two retroviruses simultaneously. In case of differential expression levels, transduction efficiencies were titrated for downstream analyses. Flow data representative for > 10 independent transductions

The mesothelin-directed T cell receptor fusion construct (MSLN-TRuC) was generated by tethering a single-chain variable fragment (scFv) derived from the anti-human mesothelin antibody SS1 to CD3ε via a flexible linker. The construct was either expressed alone in primary human T cells for HLA-independent targeting of tumor cells or co-expressed with the PD1-CD28 switch receptor. As previously described, the PD-1-CD28 comprises the extracellular and transmembrane domain of PD-1 fused to the intracellular domain of CD28 to convert a PD-1 mediated inhibitory signal into a CD28-mediated costimulatory signal (Fig. 1a and b) [28]. For co-expression of TRuC and PD-1-CD28 (TRuC + PD-1-CD28), T cells were simultaneously transduced with two separate retrovirus preparations encoding the respective constructs. The expression of the recombinant receptors was assessed by flow cytometry. Representative transduction efficiencies are shown in Fig. 1c. Transduction with MSLN-TRuC encoding retroviruses yielded 70.8% TRuC-positive cells. Similar transduction rates were achieved with the PD-1-CD28-encoding virus. Upon co-transduction, 58.3% T cells co-expressed the MSLN-TRuC and PD-1-CD28 demonstrating that a high percentage of T cells were infected by the two different viruses. Co-transduction did not affect the expression levels of the MSLN-TRuC and the PD-1-CD28 as single and double-transduced T cells expressed similar levels of TRuC and PD-1-CD28 (Fig. 1d).

### Co-expression of PD-1-CD28 increases cytokine secretion of TRuC T cells

**Fig. 2:**
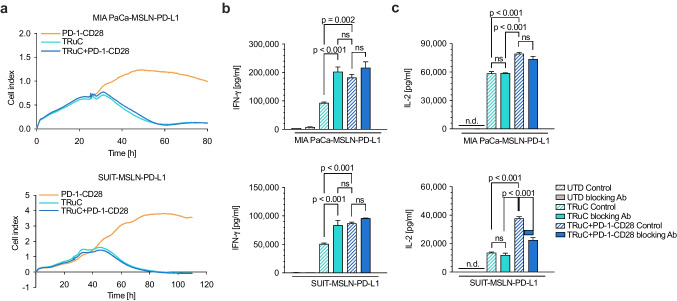
Cytokine release and killing kinetics of TRuC and TRuC + PD-1-CD28 T cells. **a**. Real-time lysis of MIA PaCa-MSLN-PD-L1 (upper panel) or SUIT-MSLN-PD-L1 tumor cells (lower panel) by transduced T cells based on impedance measurements. Effector-to-target ratio 10:1. **b**. Transduced T cells were stimulated with MIA PaCA-MSLN-PD-L1 (upper panel) or SUIT-MSLN-PD-L1 (lower panel) target cells in presence of an anti-PD-L1 blocking antibody or a corresponding isotype control antibody. Cytokine levels were quantified 48 h following stimulation using ELISA. Experiments show mean values ± SEM of duplicates and are representative of two independent experiments (two different T cell donors). For statistical analysis one-way ANOVA was used.

To study whether PD-L1 expression on tumor cells affects the effector functions of TRuC T cells, PD-L1 overexpressing SUIT-2-MSLN-PDL-1 and MIA PaCa-MSLN-PDL-1 (Supplementary Figure 1a) were co-cultured with TRuC T cells for up to 110 h. As demonstrated in a real-time impedance cytotoxicity assay, TRuC and TRuC + PD-1-CD28 T cells potently killed SUIT-MSLN-PDL-1 (Fig. 2a, upper panel) and MIA PaCa-MSLN-PD-L1 target cells (Fig. 2a, lower panel) with similar kinetics. Likewise, there was no difference in tumor cell lysis in a 24-h lysis assay using MSTO-MSLN cell kill in a 24-h lysis assay (Supplementary Figure 1b). Of note, MSLN expression is upregulated by IFN-γ which suggests that target expression in response to T cell activation is increased during the lysis assay (Supplementary Figure 1c). Compared to untransduced T cells, MSLN-TRuC T cells produce more IFN-γ after 48 h of co-culture with SUIT-MSLN-PDL-1 and MIA PaCa-MSLN-PD-L1. The production of IFN-γ is further increased in T cells that co-express the MSLN-TRuC and PD-1-CD28. Addition of a PD-L1 antibody that blocks interaction with PD-1 enhanced IFN-γ production by MSLN-TRuC T cells. These findings suggest that PD-L1 expression on tumor cells suppressed TRuC T cell activity, which could be overcome by engagement of the PD-1-CD28 with PD-L1 or the neutralizing effect of the anti-PD-L1 antibody (Fig. 2b). This suppressive effect also applies to CAR T cells, which released similar IFN-γ levels compared to TRuC T cells when stimulated with MIA Paca-MSLN-PD-L1 tumor cells (Supplementary Figure 1d). Furthermore, TRuC T cells secreted IL-2 upon co-stimulation with tumor cells and IL-2 secretion was higher in MSLN-TRuC T cells co-expressing the PD-1-CD28 (Fig. 2c). However, the neutralizing anti-PD-L1 antibody was not able to elevate IL-2 in TRuC T cells. The decreased IL-2 release by TRuC + PD-1-CD28 T cells in the presence of the anti-PD-L1 antibody is due to the lack of stimulation of the fusion protein as CD28 downstream signaling stimulates IL-2 production.

### TRuC and TRuC + PD-1-CD28 T cells show similar anti-tumor efficiency in vivo

**Fig. 3:**
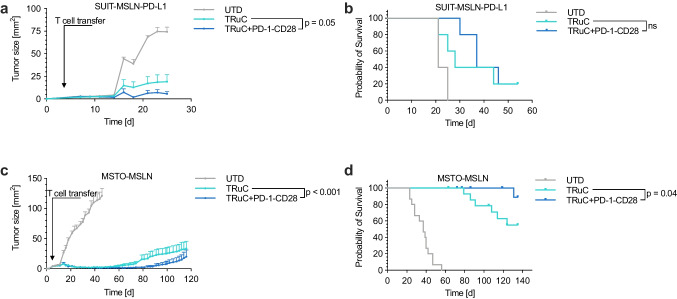
In vivo anti-tumor activity of TRuC and TRuC + PD-1-CD28 T cells. **a, b**. NSG mice were subcutaneously inoculated with SUIT-MSLN-PD-L1 target cells and treated with T cells when tumors were established. Tumor growth (**a**) and survival (**b**) was monitored for 54 days. *n* = 5 mice per group. **c**,** d**. NSG mice were subcutaneously inoculated with MSTO-MSLN target cells and treated with T cells when tumors were established. Tumor growth (**c**) and survival (**d**) were monitored for > 100 days. *n* = 15 mice for UTD or TRuC and *n* = 14 mice for TRuC + PD-1-CD28. Given the duration of the experiment (> 140 days), several mice developed GvHD and had to be censored towards the end of the experiment. Experiments show mean values ± SEM. Data shown in panel a and b were obtained by performing one single experiment with one T cell donor, panel c and d show pooled data of three independent experiments (three different T cell donors). Analyses of differences between groups were performed using unpaired, two-way Student’s t test (**a**, **c**), Log-rank test (**b**) or Gehan-Breslow-Wilcoxon test (**d**)

To further understand the consequences of our in vitro findings and to evaluate the potential of PD-1-CD28 in conjunction with TRuC T cells, we analyzed its impact in two xenograft tumor models. First, SUIT-MSLN-PD-L1 were engrafted subcutaneously, and mice were treated with transduced T cells when tumors were established. Mice injected with untransduced T cells (UTD) showed a rapid tumor growth and met the predefined experimental endpoints within 25 days following tumor cell inoculation (Fig. 3a and b). Treatment with TRuC or TRuC + PD-1-CD28 T cells slowed down tumor growth with advantages for mice treated with TRuC + PD-1-CD28 T cells. This delay in tumor growth, did however not translate in prolonged survival for mice treated with TruC + PD-1-CD28 T cells. As a reference, tumor progression in mice injected with CAR T cells was similar to TRuC T cell treated mice (Supplementary Fig. 2a). In a second subcutaneous tumor model, NSG mice were inoculated with MSTO-MSLN target cells and adoptive T cell transfer was performed when tumors were established. Injection of UTD T cells did not affect tumor growth and mice met predefined termination endpoints within 56 days after inoculation (Fig. 3c and d). Both TRuC and TRuC + PD-1-CD28 T cells were able to reduce tumor size and completely eradicate tumors. Several mice thereafter relapsed, but relapses were less frequent and slower in TRuC + PD-1-CD28 T cell treated mice resulting in prolonged survival of mice treated with TruC + PD-1-CD28 T cells compared to TRuC T cells (Fig. 3d). Due to the duration of the experiment (> 120 days) several mice in the TRuC (3/15 mice) and TRuC + PD-1-CD28 (5/14 mice) treatment group developed xeno-GVHD and were excluded from the analysis. As in the first tumor model, treatment with TRuC or CAR T cells resulted in a similar anti-tumor response, while TRuC + PD1-CD28 T cells demonstrated an advantage in tumor control (Supplementary Figure 2b), indicative of the sustained therapeutic benefit mediated by PD-1-CD28-co-expression in TRuC T cells.

## Discussion

In the present study we demonstrate that the amino-terminus of CD3ε can be modified with a mesothelin-specific scFv and that the engineered CD3ε is combined with other TCR subunits to form a functional TCR complex with novel target specificity resulting in T cell activation, cytokine release and anti-tumor activity. The co-expression of a PD-1-CD28 fusion protein antagonized PD-L1-mediated suppression of TRuC + PD-1-CD28 T cells as shown in enhanced cytokine release and improved anti-tumor activity in PD-L1 + tumor models. Together, these findings illustrate the therapeutic potential of PD-1-CD28 to further improve TRuC T cells for the treatment of PD-L1 + tumors.

In hematological and solid cancers, tumor-reactive T cells are challenged by immune-suppressive features of the tumor cells themselves as well as the tumor microenvironment. One of the major immune evasion molecules is PD-L1, which is highly expressed in various cancers and can inhibit T cell activation [30]. The interaction of PD-L1 on tumor cells and PD-1 on activated T cells similarly limits the anti-tumor capacity of endogenous tumor-reactive T cells and adoptively transferred T cells, including TRuC and CAR T cells [8]. Several strategies have already been developed to address the challenge relating to PD-1-PD-L1 induced T cell exhaustion to potentially enhance T cell function [31–33]. By sustaining an endogenous immune response, PD-1 or PD-L1 blocking antibodies have revolutionized cancer treatment and immune checkpoint blockade (ICB) has been established as a new standard of care for patients with various cancers. In a next step, ICB has been combined with CAR T cells therapy with the aim of improving CAR T cell efficiency and persistence. Early clinical reports of ICB and CD19 CAR T cell therapy in children with B-ALL indicate an improved tumor control and survival by the combination therapy [34]. A recent clinical trial combining an anti-mesothelin CAR with PD-1-blockade indicates that both agents could act synergistically at least in mesothelioma [35]. Despite these encouraging therapeutic responses, ICB exhibit the risk of autoimmune-related adverse effects by uncontrolled proliferation of self-reactive T cell clones [36, 37]. To reduce the occurrence of potentially severe side effects, novel targeted strategies must be developed to overcome tumor-induced T cell anergy and dysfunction without compromising safety. Rather than systemically blocking immune checkpoints, we therefore decided to use a PD-1-CD28 fusion protein to provide PD-L1 resistance aiming for enhanced functionality and persistence of TRuC T cells. We previously reported, that PD-1-CD28 can shield T cells from PD-L1-mediated effects and enhances the function of TCR-specific T cells [24, 27]. These results providing proof-of-concept for synergy with TCR signaling motivated the herein presented combination with the TRuC platform. A further advantage of fusion receptors over immune checkpoint blockade, PD-1 knockout or dominant-negative receptors is that inhibitory signals are not only neutralized but transformed into a T cell activating stimulus by triggering the intracellular CD28 domain. This immune-stimulatory function is demonstrated in our study by a higher activation of TRuC + PD-1-CD28 T cells in the presence of PD-L1 + tumor cells. Another strategy could be the use of a PD-1–4-1BB fusion receptor, but the PD-1-CD28 design has been shown to be superior in amending CAR T cell functionality [28, 38].

We found that PD-L1 inhibition by a monoclonal antibody enhanced cytokine production in TRuC T cells resulting in IFN-γ levels comparable to TRuC + PD-1-CD28 T cells, indicating that the advantage of TRuC + PD-1-CD28 is indeed mediated by the interaction of PD-1-CD28 with PD-L1. Similar findings have been reported for anti-CD19 CAR T cells that have been engineered to co-express a PD-1-CD28 fusion protein. By co-expression of the fusion protein, anti-CD19 CAR T cells maintained their activation potency, cytokine production and anti-leukemic function when compared to conventional anti-CD19 CAR T cells in PD-L1-positive hematological tumor models [28]. This effect of an enhanced therapeutic response by PD-1-CD28-co-expressing cells has been confirmed in our solid tumor models. In addition to improved short-term activity, fusion receptor-expressing CAR T cells were also shown to acquire a less differentiated phenotype following stimulation with PD-L1 + tumor cells, which further demonstrates their superior anti-tumor potential since less differentiated T cells are associated with advantages in proliferation and persistence relative to more differentiated T cell subsets [39, 40]. Therefore, the difference in tumor relapse in the MSLN-MSTO model might be the result of a higher proliferation and persistence of TRuC + PD-1-CD28 T cells following activation of the fusion protein. This hypothesis, however, must be confirmed in additional studies.

In a phase Ib study of patients with refractory or relapsed DLBCL after failure of anti-CD19 CAR T therapy, a single infusion of CAR T cells co-expressing a PD-1-CD28 fusion protein induced a potent anti-tumor response (3/6 complete response and 1/6 stable disease) demonstrating the potential of anti-CD19 CAR-PD-1-CD28 T cells as salvage treatment, when first CAR T therapy proves ineffective or the disease is resistant to such approach [41]. Furthermore, anti-CD19 CAR-PD-1-CD28 T cells demonstrated a manageable safety profile with patients developing grade 1–2 CRS symptoms after infusion which resolved fully by supportive treatment or administration of tocilizumab and glucocorticoids [39, 41]. A longer observation after infusion, however, is required to further assess the long-term anti-tumor efficacy of this approach. In our experiments, we observed GVHD-related symptoms in TRuC and TRuC-PD-1-CD28 T cell treated mice, which exclusively occurred in mice > 60 days post infusion. The pathogenesis of xeno-GVHD is a limitation of long-term mouse xenograft models which is induced by human T cells that recognize murine xeno-antigens presented on murine MHC and interact with murine B7.2 molecules, and therefore does not have any known pathophysiological correlate in humans [42, 43]. Our conclusion is further supported by the observation that currently available preclinical xenograft mouse models are poorly predictive of the clinical toxicity of CAR T cells owing to the lack of bystander human hematopoiesis and therefore lack of CRS development (monocytes rather than CAR T cells are primarily responsible for the systemic release of IL-6 which ultimately causes CRS). This issue has been addressed by Norelli et al. who recently established a novel xeno-tolerant mouse model, which allows to investigate long-lasting CAR-mediated effects including CRS and neurotoxicity [43]. Overall, the favorable clinical safety profile of anti-CD19 CAR-PD-1-CD28 T cells is thought to be due to the local activation of the fusion protein specifically in the tumor environment. This local effect could be further stimulated by the additional modification of PD-1-CD28-expressing CAR or TRuC T cells with a chemokine receptor that enhances the accumulation of the engineered T cells in the tumor tissue [1, 11, 44, 45]. This combination of receptors might be advantageous for the treatment of solid tumors, where tumor infiltration (besides immunosuppression) is a major factor limiting the efficacy of adoptively transferred T cells. The chemokine receptor-mediated tumor trafficking combined with the shielding effect of the PD-1-CD28 fusion protein could have the potential to overcome these limitations and extend the therapeutic success of CAR or TRuC T cells to solid malignancies.

Recently, CAR T cells with PD-1 (PDCD1) knockout showed an increased capacity in controlling tumor growth in xenograft models, which was superior to the combination of conventional CAR T cell treatment with PD-1 antibody blockade [32, 46]. However, PD-1 depletion has been associated with unrestricted T cell growth and was found to be a tumor suppressor in T cell lymphoma [47]. Such results call to attention that while gene editing might be a powerful method to disrupt genes in T cells, these changes might also come with a heavy toll to the treated patients, in the event of potential oncogenic events. Although preliminary data of a dose-escalation study in patients with mesothelin-positive solid tumors showed feasibility and tolerable safety profile of CAR T cells with PDCD1 disruption [22], long-term studies are required to evaluate the effect of PD-1 depletion on expansion and persistence of CAR T cells. Interestingly, a phase I clinical trial of CRISPR-Cas9 engineered T cells found a decrease in the frequency of cells with edits in the PDCD1 locus within 4 months post infusion, which may suggest that PD-1 deficient T cells are less able to establish memory features [48]. Along these lines, the PD-1-CD28 fusion protein strategy might come with a better safety and potency profile compared to the knockout of PDCD1 since the endogenous gene locus remains intact and the fusion receptor only functions as a co-stimulatory protein depending on CAR or TRuC signaling. Additionally, the fusion receptor not only depletes the immune-inhibitory signal but transforms it into a T cell-activating signal. Future clinical studies will be needed to evaluate the therapeutic benefit of PD-1-CD28 fusion protein expression in TRuC but also CAR T cells.

## Supplementary Information

Below is the link to the electronic supplementary material.

Supplementary file1 Supplementary Figure 1: Mesothelin and PD-L1 expressed on tumor cells stimulate transduced T cells. **a.** SUIT and MIA PaCa tumor cells were engineered to express the tumor antigen mesothelin (left panel) and the co-inhibitory factor PD-L1 (right panel). Representative flow data for the expression of both proteins are shown. **b.** Transduced T cell were stimulated with MSTO-MSLN and target cell lysis was quantified by measuring LDH release after 24 h. Effector-to-target ratio 5:1. **c.** MSTO tumor cells were engineered to express the tumor antigen mesothelin (left panel). Stimulation with recombinant IFN-γ induced endogenous PD-L1 in MSTO-MSLN (right panel). Representative flow data are shown. **d**. Transduced T cell were stimulated with MIA PaCa-MSLN-PD-L1 tumor cells and IFN-γ in supernatants was quantified by ELISA. Effector-to-target ratio 5:1. Experiments show mean values ± SEM and are representative of two independent experiments (a, c, d) or independent experiments with two different T cell donors (b). For statistical analysis the one-way ANOVA method was used. Supplementary Figure 2: In vivo anti-tumor activity of CAR, TRuC and TRuC + PD-1-CD28 T cells. **a.** NSG mice were subcutaneously inoculated with SUIT-MSLN-PD-L1 target cells and treated with T cells when tumors were established. Tumor growth was monitored for 54 days. n = 5 mice per group. **b.** NSG mice were subcutaneously inoculated with MSTO-MSLN target cells and treated with T cells when tumors were established. Tumor growth was monitored for > 100 days. *n* = 15 mice for UTD or TRuC, *n* = 12 mice for CAR and n = 14 mice for TRuC + PD-1-CD28. Given the duration of the experiment (> 140 days), several mice developed GvHD and had to be censored towards the end of the experiment. Experiments show mean values ± SEM. Data shown in panel a were obtained by performing one single experiment with one T cell donor, panel b shows pooled data of three independent experiments (three different T cell donors). Analyses of differences between groups were performed using unpaired, two-way Student’s t test (PDF 210 kb)

## Data Availability

The data that support the findings of this study are available from the corresponding author upon reasonable request.
