# Supplementary figures and images for: PD-1-CD28 fusion protein strengthens mesothelin-specific TRuC T cells in preclinical solid tumor models
Source: Cell Oncol (Dordr). 2022 Nov 21;46(1):227–35. doi: 10.1007/s13402-022-00747-9 (PMC9947055; doi:10.1007/s13402-022-00747-9)

# Supplementary Figure 1

**a**

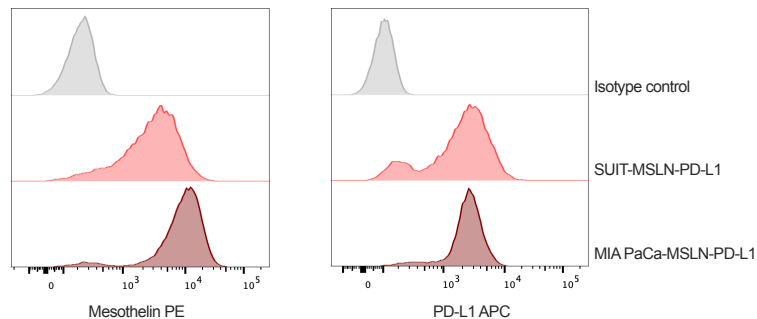

**b**

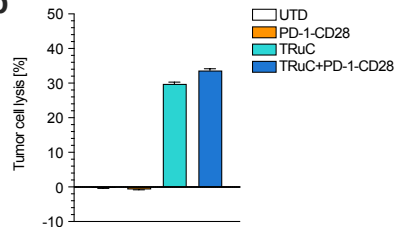

**c**

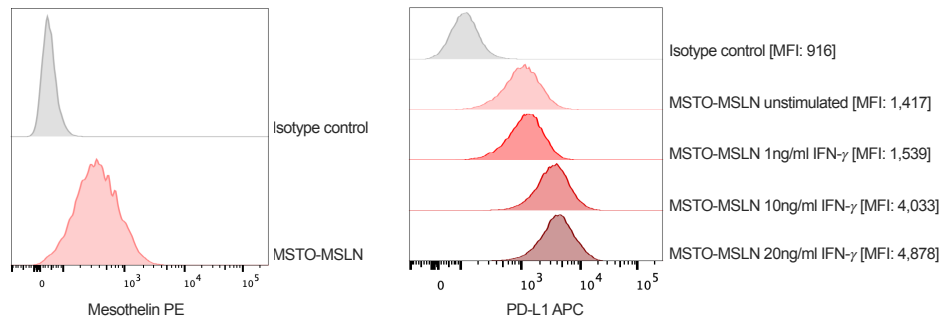

**d**

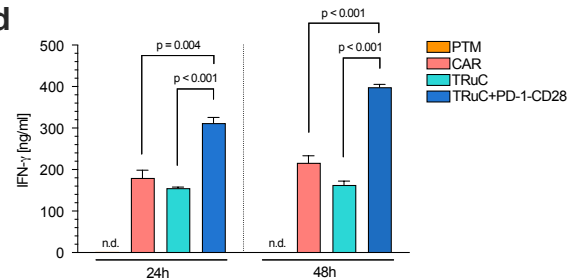

## Supplementary Figure 2

**a**

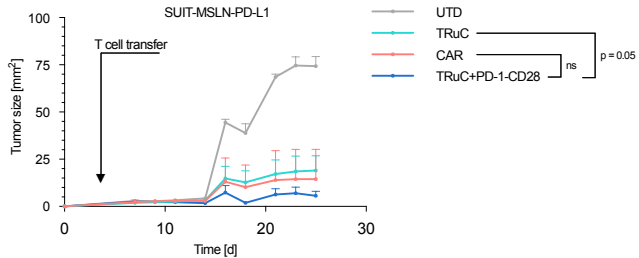

**b**

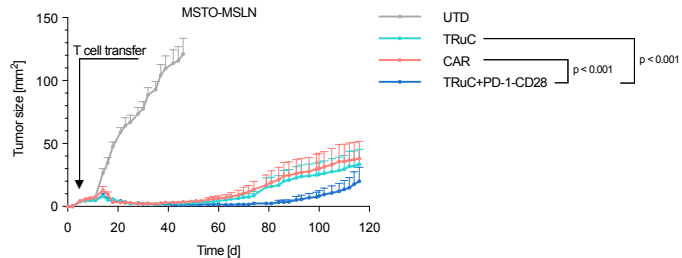

Supplement: Supplementary file 1 — Supplementary file1 Supplementary Figure 1: Mesothelin and PD-L1 expressed on tumor cells stimulate transduced T cells. a. SUIT and MIA PaCa tumor cells were engineered to express the tumor antigen mesothelin (left panel) and the co-inhibitory factor PD-L1 (right panel). Representative flow data for the expression of both proteins are shown. b. Transduced T cell were stimulated with MSTO-MSLN and target cell lysis was quantified by measuring LDH release after 24 h. Effector-to-target ratio 5:1. c. MSTO tumor cells were engineered to express the tumor antigen mesothelin (left panel). Stimulation with recombinant IFN-γ induced endogenous PD-L1 in MSTO-MSLN (right panel). Representative flow data are shown. d. Transduced T cell were stimulated with MIA PaCa-MSLN-PD-L1 tumor cells and IFN-γ in supernatants was quantified by ELISA. Effector-to-target ratio 5:1. Experiments show mean values ± SEM and are representative of two independent experiments (a, c, d) or independent experiments with two different T cell donors (b). For statistical analysis the one-way ANOVA method was used. Supplementary Figure 2: In vivo anti-tumor activity of CAR, TRuC and TRuC + PD-1-CD28 T cells. a. NSG mice were subcutaneously inoculated with SUIT-MSLN-PD-L1 target cells and treated with T cells when tumors were established. Tumor growth was monitored for 54 days. n = 5 mice per group. b. NSG mice were subcutaneously inoculated with MSTO-MSLN target cells and treated with T cells when tumors were established. Tumor growth was monitored for > 100 days. n = 15 mice for UTD or TRuC, n = 12 mice for CAR and n = 14 mice for TRuC + PD-1-CD28. Given the duration of the experiment (> 140 days), several mice developed GvHD and had to be censored towards the end of the experiment. Experiments show mean values ± SEM. Data shown in panel a were obtained by performing one single experiment with one T cell donor, panel b shows pooled data of three independent experiments (three different T cell donors). An [file 13402_2022_747_MOESM1_ESM.pdf]
